# A dataset of *in vitro* aroma release kinetics performed with proton transfer reaction-time of flight- mass spectrometry (PTR-ToF-MS)

**DOI:** 10.1016/j.dib.2026.112994

**Published:** 2026-06-25

**Authors:** I. Andriot, D. Grossiord, N. Béno, T. Chabin, H. Labouré, G. Lucchi, C. Martin, O. Mourabit, J. Piornos, C. Salles, I.C. Trelea, C Peltier

**Affiliations:** a Université Bourgogne Europe, Institut Agro, CNRS, INRAE, UMR CSGA, 21000 Dijon, France; b Probe Research Infrastructure, Chemosens facility, CNRS-INRAE, Dijon, France; c Université Paris-Saclay, INRAE, AgroParisTech, UMR SayFood, F-91120, Palaiseau, France; d Centre for Taste and Feeding Behavior, Dijon, France

**Keywords:** Mass spectrometry, PTR-MS, Temperature, Aroma compounds

## Abstract

The study of aroma release is essential in food science to better understand flavour perception. Analytical techniques such as APCI-MS and PTR-MS allow real-time monitoring of volatile compounds, including isoamyl acetate and diacetyl, directly in food systems. *In vitro* models are particularly useful because they enable controlled studies of parameters like temperature or container volume, which are difficult to isolate in real consumption conditions.

In parallel, aroma modelling has been developed to predict release kinetics under different conditions. Early mechanistic approaches focused on describing mass transfer processes governing aroma behaviour after a disturbance, such as opening a container. These models have since been extended to more complex food matrices to better simulate real systems.

This paper presents data on the kinetics of *in vitro* aroma release for two volatile compounds, in aqueous solution or incorporated into gelatin discs, measured using PTR-ToF-MS. A total of 84 experiments were conducted under varying conditions, including temperature, initial concentration or duration of preliminary equilibrium.

The dataset includes raw PTR-ToF-MS time-series data in .csv format, together with associated metadata describing the experimental conditions, analysed products, target compounds, and instrumental parameters required for data interpretation and reuse. This dataset can be used for:

- documenting PTR-ToF-MS measurements under multiple conditions initiating a database for modelling aroma release,

- relating signal intensity and real concentration,

- tracking ion and fragment relationships.

Specifications Table

**Table:** 

Subject	Biology
Specific subject area	This aim was to simulate a flavor release in the mouth with *in vitro* experiments
Type of data	Tables
Data collection	The data were collected with PTR-ToF-MS and then pre-processed in successive R and Python pre-processing software. Based on the raw .h5 files, the first pre-processing step consisted in extracting the datas expressed in counts per seconds (ion signal intensities) of PTR-ToF-MS with PTRMSR package (code available in GitHub). This pre-processing was performed using a table of integration for each ion. The second preprocessing step consisted in pooling the results of all experimentations.
Data source location	The data were collected in the Center of Taste and Feeding Behavior in Dijon (UMR CSGA, 17 rue Sully, 21,000 Dijon, France). The raw data (.h5 files) are stored locally in a shared repositoy and in a datacenter. This paper makes available the preprocessed datasets.
Data accessibility	Repository name: https://entrepot.recherche.data.gouv.fr/dataverse/chemosens Data identification number: https://doi.org/10.57745/GVXDND Direct URL to data: https://entrepot.recherche.data.gouv.fr/dataset.xhtml?persistentId=doi:10.57745/GVXDND
Related research article	None

## 1 Value of the Data

• **Documentation of PTR-ToF-MS measurements under multiple conditions:** These data record proton transfer mass spectrometry (PTR-ToF-MS) signals across several controlled experimental conditions and products, enabling researchers to compare instrument responses and detection patterns under different settings.
• **Tracking ion and fragment relationships:** The dataset captures information on target ions and their corresponding fragments, providing a reference for studies on ion behaviour and method development in mass spectrometry.
• **Relating signal intensity and real concentration:** Measurements using H_3_O^+^ and H_3_O^+^(H_2_O) ions allow examination of signal strength and relative concentrations, supporting studies of ionization efficiency and analyte quantification. Thus, using different types of corrections (using the H_3_O^+^) could be compared.
• **Initiating a database for modelling aroma release:** The protocol and data used in this paper could be completed by other experimentations with other aroma, matrices or conditions. It could pave the way for a database on aroma release kinetics.
• These data will be useful for researchers working in mass spectrometry, food science, flavour chemistry, especially flavour scientists working with PTR-MS. They may support the development of PTR-ToF-MS methodologies, improve the interpretation of ionization and fragmentation processes, and provide reference data for studies investigating aroma release and its modelling under different experimental conditions.

## 2 Background

Following aroma release is of major interest in food science, as it provides key insights into flavor [1]. Real-time monitoring of aroma release is now possible using advanced analytical techniques such as Atmospheric Pressure Chemical Ionization Mass Spectrometry (APCI-MS), as well as Proton Transfer Reaction Mass Spectrometry (PTR-MS) [2], which can be applied directly to real food systems. In particular, *in vitro* systems allow the investigation of a wide range of controlled conditions, such as temperature and vial volume, that are much more difficult to reproduce *in vivo*.

In parallel, aroma modelling has emerged as a promising approach to predict aroma release kinetics across systems and conditions [3,4]. Early work focused on establishing the core mass transfer mechanisms governing aroma release *in vitro*, using mechanistic models to describe how volatile profiles evolve when equilibrium is disrupted, such as when a food container is opened [4]. Building on these principles, further mechanistic models aimed to predict aroma release from more complex matrices.

Within this context, high-quality experimental datasets are required to support aroma release modelling. The dataset presented in this paper consists of 84 *in vitro* release profiles obtained using PTR-MS under various experimental conditions from aqueous solutions of isoamyl acetate and diacetyl, as well as from controlled gel-based model systems.

## 3 Data Description

This article describes the data of aroma release kinetics during several PTR-ToF-MS acquisitions collected in 2025, April and May by the UMR CSGA (Dijon, France).

It consists of three datasets:

• sample_metadata (sample_metadata_final.csv) describes each sample and its associated experimental conditions.
• var_metadata (var_metadata.tab contains information about the different variables measured (the ions), including their name, *m/z*, and the lower and upper bounds used in data preprocessing.
• total_data.tab contains the data obtained from 84 experiments.

**Description of the sample_metadata dataset:**

This dataset contains 84 aroma release kinetics whose variables are described in Table 1. For each tested conditions, three replicates are available. Further instrumental parameters are available such as the PTR-MS flow rate. Table 1 describes exhaustively the columns of sample_metadata dataset.

**Table 1:** Variables in the sample_metadata dataset.

Column	Description	Values
temperature (°C)	Measurement temperature (°C)	22 or 36
equilibrium_time (min)	Equilibration time before measurement (min)	5, 10, 15 or 20
ptr_flow_rate (ml/min)	PTR instrument aspiration flow rate (mL/min)	158
vial_volume (ml)	Sample container volume (mL)	30 or 50
start (s)	Measurement start time (s)	30
stop (s)	Measurement stop time (s)	150
c0	Initial concentration of the analyte or reference compound	2 or 0.4
Unit	Unit of concentration or signal.	‘ppm’ or ‘g/L’
vol (mL)	Sample volume used for the measurement (mL)	2
Agit	Agitation conditions during the measurement (*e.g.*,”)	stirred =”180 ” (rpm or static=”no
Type	Type of sample or experiment	(“sol” for solution, “gel” for gelatin discs)
v_balay (ms)	Sweep or scan time of the instrument (ms)	100 or 500
Date	Date of measurement	-
order	Order of sample analysis or injection sequence	-
molecule	Name of the molecule or compound measured	‘ACI’ (for isoamylacetate) or ‘diacetyl’

**Description of the var_metadata dataset:**

Six ions were monitored during all the experiments: isoamyl acetate (C_7_H_15_O_2_^+^, *m/z*131), *m/z*71 (C_5_H_11_^+^) and *m/z*61 (C_2_H_5_O_2_^+^) (fragment of isoamyl acetate that are fragments produced by the ionization step in PTR-MS), diacetyl (C_4_H_7_O_2_^+^, m/87), H_3_^18^O^+^ (*m/z* 19) and H_3_^18^O^+^(H_2_O)(*m/z*39). For each ion, var_metadata contains the name of aroma (used in data_matrix), theoretic *m/z* of these ions, and two values: ‘inf’ and ‘sup’ that indicates in which interval of *m/z* the collected data correspond to the ion. Finally, the ion name is provided. These values are used during the preprocessing of the data. The last column contains the CAS (Chemical Abstracts Service) number, a unique numerical identifier, for each molecule.

**Description of the total_data:** total_data is stored in a long format with 5 columns: time, intensity, duration, ion and file. File contains the names of the 84 files described in the sample_metadata file. Time and duration are in seconds. ‘ion’ is the name of the studied ion. It is related to a var_metadata file. Ion signal intensities were recorded in counts per second, named Intensity in the table of integration at each timepoint for each ion. This intensity was obtained running a R code with var_metadata as integration table [5]. The code used to produce the data frame is freely available on GitHub. https://github.com/Chemosens/ExternalCode/tree/main/Digimouth/preprocessingPTRMS)

## 4 Experimental Design, Materials and Methods

### 4.1 Preparation of samples

#### 4.1.1 Preparation of solutions

A 2 g.L^−1^ stock solution of isoamyl acetate was prepared by dissolving 200 mg of isoamyl acetate in 100 mL of Evian^Ⓡ^ water. The stock solution was stirred continuously for 2 h at room temperature to ensure complete dissolution. A 0.4 g.L^−1^ working solution was then prepared by diluting 20 mL of the stock solution to a final volume of 100 mL with Evian^Ⓡ^ water. Then, a working solution was prepared at 2 mg.L^−1^ in Evian^Ⓡ^ water, by diluting 500 µl of solution at 0.4 g. L^−1^ to a final volume of 100 mL with Evian^Ⓡ^ water. A stock solution of diacetyl at 34.6 mg.L^−1^ was prepared by dissolving 34.6 mg of diacetyl in Evian^Ⓡ^ water and adjusted at 100 mL with Evian^Ⓡ^ water. The solution of diacetyl at 2 mg.L^-1^ in Evian^Ⓡ^ water was prepared, by diluting 290 µl of stock solution to a final volume of 100 mL with Evian^Ⓡ^ water.

#### 4.1.2 Preparation of gelatin discs

Gelatin discs were prepared using 45 g of commercial pork gelatin powder (Vahiné^Ⓡ^) and 200 g of Evian^Ⓡ^ water. The gelatin powder was hydrated in Evian^Ⓡ^ water for 5 min. An additional 150 g of Evian^Ⓡ^ water, preheated in a microwave for 1 min, was then added. The mixture was homogenized using a Thermomix and cooled to 37 °C while gently mixing. Once the target temperature was reached, 100 g of the 2 g/L isoamyl acetate solution was incorporated, and the mixture was homogenized for an additional 2 min. The final mixture was transferred into 2 mL portions in silicone molds (Flexipan^Ⓡ^) using a pipette. The molds were stored at 4 °C until complete gelation occurred for approximatively 12 h. The fully gelatin discs were then transferred into individual closed containers for storage.

### 4.2 Instrumental

**Fig. 1:**
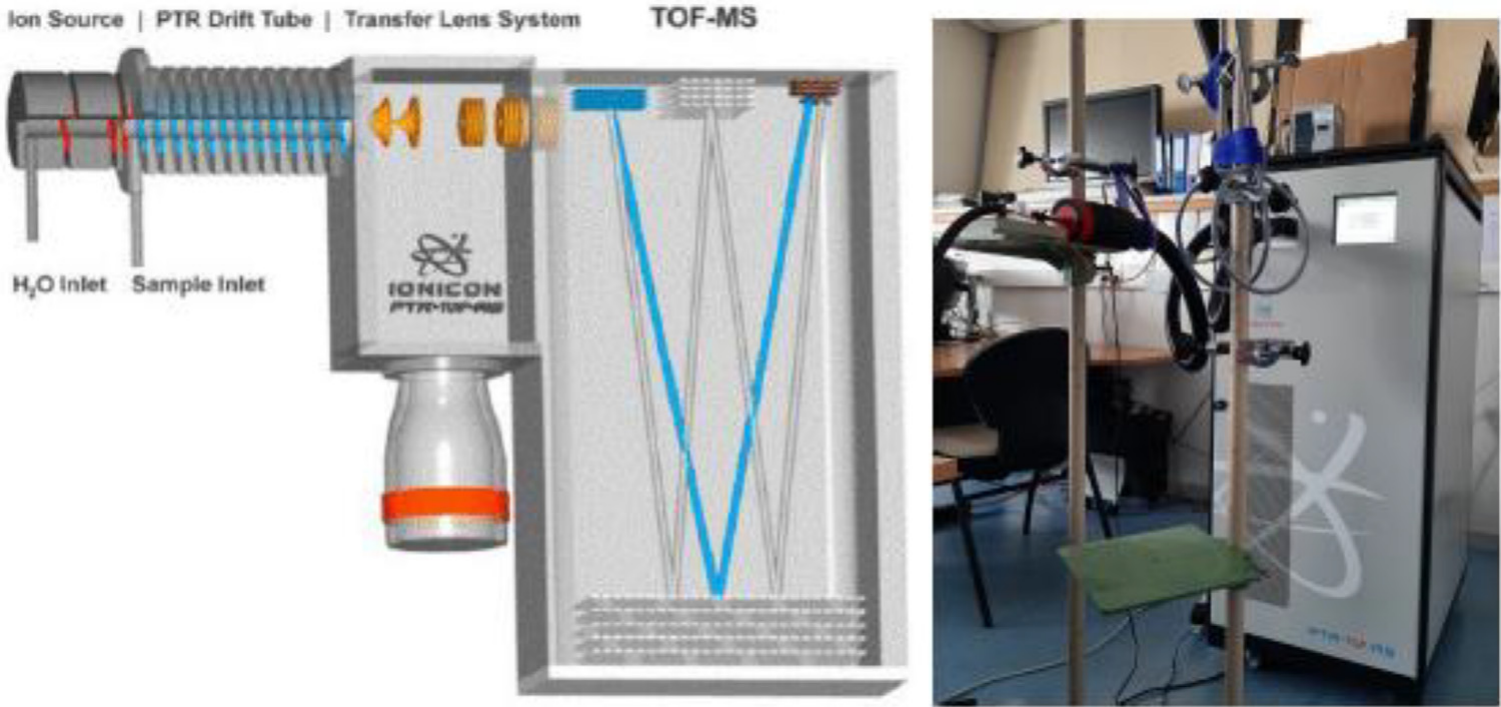
Schematic diagram of the PTR-ToF-MS and photograph of the PTR-ToF-MS used (PTR-ToF 8000, Ionicon, Analytik GmbH, Austria).

The PTR-ToF-MS (Proton Transfer Reaction–Time-of-Flight Mass Spectrometry; PTR-ToF 8000, Ionicon Analytik GmbH, Innsbruck, Austria), equipped with an ion funnel to improve ion focusing into the ToF mass analyzer, was used to ionize volatile compounds via proton transfer from H₃O⁺ (Fig. 1).

The parameters of the PTR-ToF-MS instrument were as follows: drift pressure of 2.3 mBar, drift temperature of 80 °C, and drift voltage of 390 V, resulting in an electric field strength to number density ratio (E/N ratio) of 115 Townsend (Td, 1 Td = 10^−17^ V.cm^2^). The temperature of the transfer line of the PTR-MS was maintained at 110 °C. Data were collected using the TofDAQ software provided by the manufacturer of the PTR-ToF-MS. Data acquisitions were performed at 1 mass spectrum ranging from *m/z* 0 to227 per 100 ms.

### 4.3 Experimental design

**Fig. 2:**
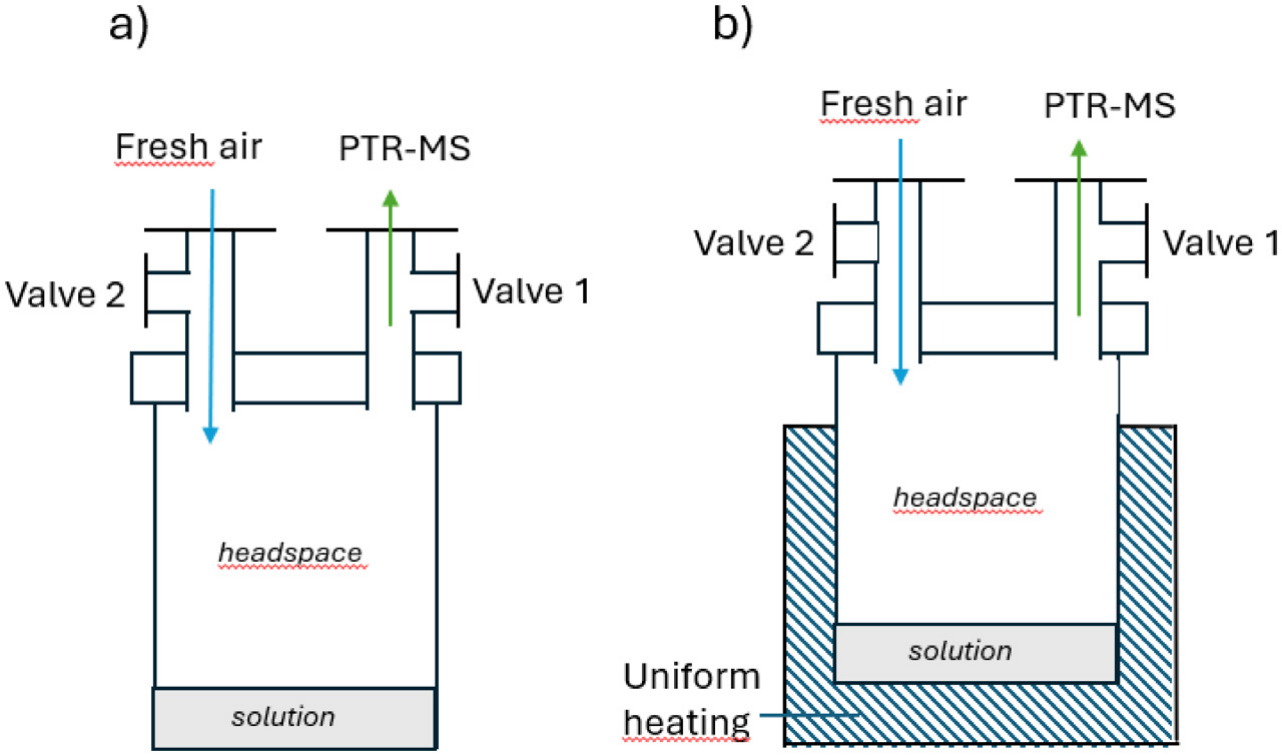
Vials used for the headspace analyses a) without heating b) with heating.

First, the product was placed in a vial (30 or 50 mL). The headspace was then allowed to equilibrate for 5, 10, 15, or 20 min. Headspace analyses were performed either at room temperature or at 36 °C to mimic oral conditions. When required, the vials were maintained at 36 °C using a heating block, and the temperature was monitored throughout the experiment with a thermometer. Note that our device could contain several vials: one vial was used specifically to measure the temperature, and the second one for measuring the headspace with PTR-MS. The vials were connected to the transfer line of PTR-MS, by a flexible heated (75 °C) PEEK tubing (85 cm long, 1 mm internal diameter). The headspace formed in the upper part of the vials was drawn into the PTR-MS. A valve located on the vial cap allowed ambient air to enter the vial, thereby preventing the formation of a vacuum (Fig. 2). Valve 1 was connected to the PTR-ToF-MS and collected the analyzed sample, while Valve 2 allowed ambient air to enter the vial, preventing the formation of a vacuum.

Each experiment lasted 3 min: 30 s for the blank (*i.e.*, ambient air), 2 min for the vial headspace, and 30 s for the final blank.

## Limitations

Users of this dataset should consider the following limitations. First, the final concentration of isoamyl acetate in the gelatin discs systems was not directly measured; concentration values are only available for the initial solutions. Consequently, the measured signal intensities should be interpreted in relative terms rather than as absolute quantitative values.

Second, partial evaporations of the solutions may have occurred during repeated bottle openings, potentially leading to variations in maximum signal intensity between experiments. However, this effect is not expected to impact the aroma release kinetics, but only its intensity. For this reason, the order of sample presentation is provided to allow users to account for potential intensity drift when analyzing the data.

## CRediT Author Statement

**Isabelle Andriot**: Conceptualization, Methodology, Project administration, Visualisation, Writing – original draft. **Caroline Peltier**: Initiation, Conceptualization, Methodology, Project administration, Writing – original draft –. **Cristian Trelea:** Conceptualization, Methodology, Project administration. –. **Danaé Grossiord**: Investigation**,** Conceptualization, Methodology –. **Noelle Béno**: Methodology, Writing – review and editing –. **Thibault Chabin**: Methodology, Writing – review and editing. **Hélène Labouré**: Methodology, Writing – review and editing –. **Géraldine Lucchi:** Methodology, Writing – review and editing –. **Christophe Martin**: Methodology, Writing – review and editing –. **José Piornos:** Methodology, Writing – review and editing –. **Christian Salle**s: Methodology, Writing – review and editing –. **Kipédène Coulibaly;** Methodology, Writing – review and editing.

## Ethics Statement

Not applicable.

## Declaration of Generative AI and AI-Assisted technologies in the Manuscript preparation Process

During the preparation of this work, the author(s) used ChatGPT for English correction. The author(s) reviewed and edited the output as needed and take full responsibility for the content of the published article.

## Data Availability

DataverseDataset of aroma release in vitro (Digimouth) (Original data)
DataverseDataset of aroma release in vitro (Digimouth) (Original data)
